# An Unusual Giant Leg Ulcer as a Rare Presentation of Sweet’s Syndrome in a Patient with Hairy Cell Leukemia Successfully Managed by Splenectomy

**DOI:** 10.4274/tjh.2016.0416

**Published:** 2017-08-02

**Authors:** Hakan Özdoğu, Mahmut Yeral, Can Boğa

**Affiliations:** 1 Başkent University Faculty of Medicine, Department of Hematology, Ankara, Turkey

**Keywords:** Hairy cell leukemia, splenectomy, Sweet’s syndrome

## To The Editor,

A 56-year-old male patient who was known to be diagnosed with hairy cell leukemia (HCL) and went into remission with cladribine 7 years ago was admitted to our clinic due to anemia and a leg ulcer.

**Figure 1:**
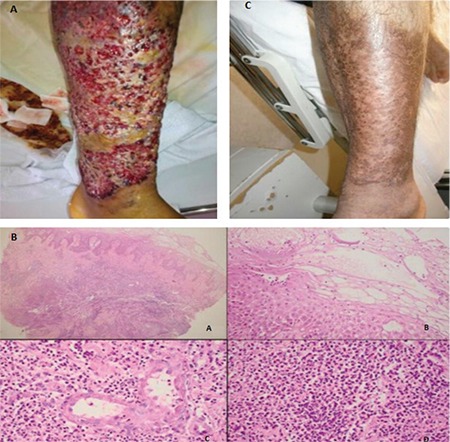
A giant ulcer greater than 20 cm in size with an irregular margin and tender and hyperemic skin changes arising adjacent to the ulcer. B) Photomicrograph reveals the histopathologic details of the skin biopsy. The inflammatory infiltration was dense and extended to the subdermis (A). There was edema, spongiosis, and neutrophil and eosinophil infiltration of the epidermis (B). Dermal infiltration was composed of eosinophil polymorphs and neutrophil polymorphs (C). Microabscess formation was observed (D) [A: Hematoxylin and eosin (H&E), 40^x^, B: H&E, 200^x^, C: H&E, 200^x^, D: H&E, 200^x^]. C) Significant improvement of leg ulcer 2 months after splenectomy.

One month before his admission, the patient developed weakness and exertional dyspnea. On his admission, relapsed leukemia with a giant ulcer with irregular borders on the anterior side of the left thigh, present for the past 20 days, was diagnosed (Figure 1A). At the time of admission, hemoglobin was 7.6 g/dL and platelet count was 39x10^9^/L. The leukocyte count was 1.4x10^9^/L with a dominance of lymphocytes (76%). Microscopic examination of the marrow and immunophenotyping of lymphocytes revealed HCL with characteristic morphology and positivity of hairy cell markers like CD25, CD103, and CD11c. Infiltration rate with hairy cells was 80%. Meanwhile, no environmental causes of an ulcer like an accident, drug use, or chemical exposure were identified. The bacterial culture taken from the ulcerous surface was negative.

Microscopic examination of the skin biopsy showed hyperkeratosis, parakeratosis, acanthosis, edema, spongiosis, necrotic keratinocytes, eosinophil and neutrophil exocytosis in the epidermis, and a dense inflammatory infiltration extending into the subdermal layer. The inflammatory infiltrate was composed of eosinophil polymorphs and neutrophil polymorphs. Skin biopsy findings were consistent with Sweet’s syndrome (Figure 1B).

While cladribine and pentostatin are known as first treatment options for relapsed/refractory HCL, there is not a consensus about the use of rituximab in those cases [1]. Therefore, our patient underwent splenectomy. The ulcer healed rapidly and hematological parameters improved within weeks after the splenectomy (Figure 1C). The post-splenectomy complete blood count revealed hemoglobin of 11 g/dL, leukocyte count of 8.6x10^9^/L with 60% lymphocytes, and platelet count of 153x10^9^/L. Three months after the splenectomy, when the leg ulcer had healed completely, the patient could receive cladribine (0.1 mg/kg/day continuous infusion for 7 days) successfully to treat the disease. Flow cytometric analysis yielded negative minimal residual disease in the bone marrow after cladribine. He remains leukemia-free at 24 months of follow-up.

The giant leg ulcer in the presented case was uncommon and seemed dramatic. In the involved skin, the absence of leukemic infiltration, evidence of an infection, or vasculitis pointed towards Sweet’s syndrome with typical histological findings.

Several studies have indicated a link between HCL and Sweet’s syndrome [2,3]. Although the immunological mechanism is not completely defined for Sweet’s syndrome, a chemoattractive substance released from leukemic cells may play a role in developing neutrophilic tissue infiltration. These substances can be attributed to IL-8, leukocyte function antigen, or gamma interferon [2,3,4]. For this reason, for complete healing, this lesion required resolution of the underlying leukemia. Naturally, the patient was at high risk of infection and tissue damage because of the ulcer, so he could not receive cladribine for the treatment of HCL. Splenectomy was applied to control the leukemic burden. This procedure provided leukemia control without using any chemotherapeutic drugs. The leukemia went into hematological remission and the ulcer size gradually decreased within a few months.

Splenectomy is a historical treatment approach in HCL [5]. This treatment nonetheless may still be utilized in patients with relapsed or refractory disease. The rational for splenectomy is the minimizing of tumor burden. In addition, it has been reported that splenectomy may lead to a decrease in the level of some chemokines or cytokines [6,7]. The mechanisms mentioned above might be responsible for controlling Sweet’s syndrome following splenectomy.

In conclusion, this case reminds us that uncommon manifestations may develop in HCL. If so, to control those abnormalities, it may be necessary to return to historic treatment strategies in situations limiting chemotherapy.
